# Effect of a Novel Online Group-Coaching Program to Reduce Burnout in Female Resident Physicians: A Randomized Clinical Trial

**DOI:** 10.1001/jamanetworkopen.2022.10752

**Published:** 2022-05-06

**Authors:** Tyra Fainstad, Adrienne Mann, Krithika Suresh, Pari Shah, Nathalie Dieujuste, Kerri Thurmon, Christine D. Jones

**Affiliations:** 1 Division of General Internal Medicine, Department of Medicine, University of Colorado Anschutz Medical Campus, Aurora; 2 Lowry Internal Medicine, Denver, Colorado; 3 Division of Hospital Medicine, Department of Medicine, University of Colorado Anschutz Medical Campus, Aurora; 4 Rocky Mountain Regional Veterans Affairs Medical Center, Aurora, Colorado; 5 Department of Biostatistics and Informatics, Colorado School of Public Health, Aurora; 6 Adult and Child Consortium for Health Outcomes Research and Delivery Science, University of Colorado, School of Medicine, Aurora; 7 Graduate School of Social Work, University of Denver, Denver, Colorado; 8 Division of Urology, Department of Surgery, Denver Health, Denver, Colorado; 9 Veterans’ Health Administration, Eastern Colorado Health Care System, Denver-Seattle Center of Innovation for Veteran-Centered and Value Driven Care, Aurora

## Abstract

**Importance:**

Female resident physicians are disproportionately affected by burnout, which can have serious consequences for their well-being and career trajectory. Growing evidence supports the use of professional coaching to reduce burnout in resident physicians, yet individual coaching is resource intensive and infeasible for many training programs.

**Objective:**

To assess whether a structured professional group-coaching program for female resident physicians would lead to decreased burnout.

**Design, Setting, and Participants:**

This pilot randomized clinical trial was conducted from January 1 to June 30, 2021, among 101 female resident physicians in graduate medical education at the University of Colorado who voluntarily enrolled in the trial after a recruitment period. Surveys were administered to participants before and after the intervention.

**Intervention:**

With the use of a computer-generated 1:1 algorithm, 50 participants were randomly assigned to the intervention group and 51 participants were randomly assigned to the control group. The intervention group was offered a 6-month, web-based group-coaching program, Better Together Physician Coaching, developed and facilitated by trained life coaches and physicians. The control group received residency training as usual, with no coaching during the study. The control group was offered the 6-month coaching program after study completion.

**Main Outcomes and Measures:**

The primary outcome of burnout was measured using the Maslach Burnout Inventory, defined by 3 Likert-type 7-point subscales: emotional exhaustion, depersonalization, and professional accomplishment. Higher scores on the emotional exhaustion and depersonalization subscales and lower scores on the professional accomplishment subscale indicate higher burnout. Secondary outcomes of impostor syndrome, self-compassion, and moral injury were assessed using the Young Impostor Syndrome Scale, Neff’s Self-Compassion Scale–Short Form, and the Moral Injury Symptom Scale–Healthcare Professionals, respectively. An intention-to-treat analysis was performed.

**Results:**

Among the 101 female residents in the study, the mean (SD) age was 29.4 (2.3) years, 96 (95.0%) identified as heterosexual, and 81 (80.2%) identified as White. There were 19 residents (18.8%) from surgical subspecialties, with a range of training levels represented. After 6 months of professional coaching, emotional exhaustion decreased in the intervention group by a mean (SE) of 3.26 (1.25) points compared with a mean (SE) increase of 1.07 (1.12) points in the control group by the end of the study (*P* = .01). The intervention group experienced a significant reduction in presence of impostor syndrome compared with controls (mean [SE], −1.16 [0.31] vs 0.11 [0.27] points; *P* = .003). Self-compassion scores increased in the intervention group by a mean (SE) of 5.55 (0.89) points compared with a mean (SE) reduction of 1.32 (0.80) points in the control group (*P* < .001). No statistically significant differences in depersonalization, professional accomplishment, or moral injury scores were observed. Owing to the differential follow-up response rates in the treatment groups (88.2% in the control group [45 of 51]; 68.0% in the intervention group [34 of 50]), a sensitivity analysis was performed to account for the missing outcomes, with similar findings.

**Conclusions and Relevance:**

In this randomized clinical trial, professional coaching reduced emotional exhaustion and impostor syndrome scores and increased self-compassion scores among female resident physicians.

**Trial Registration:**

ClinicalTrials.gov Identifier: NCT05280964

## Key Points

**Question:**

Can a 6-month online group-coaching program targeted for various learning styles reduce burnout, moral injury, and impostor syndrome and increase self-compassion among female resident physicians?

**Findings:**

In this pilot randomized clinical trial of 101 female resident physicians, participants who were randomly assigned to a 6-month group-coaching program and a follow-up survey had a statistically significant reduction in the emotional exhaustion subscale of burnout compared with the control group.

**Meaning:**

An online multiformat group-coaching program may be an effective intervention to decrease burnout and improve well-being for female resident physicians.

## Teaser

This pilot randomized clinical trial assesses whether a structured professional group coaching program for female resident physicians would lead to decreased burnout.

## Introduction

Burnout, referring to feelings of exhaustion, negativism, and reduced personal efficacy at work, affects 25% to 30% of individuals in the US and 44% to 80% of medical trainees and physicians.^1^ Physician burnout is associated with increased errors, higher patient mortality rates, depression, suicidal ideation, and high job turnover.^2,3^ Physician burnout has been described as a “public health crisis that urgently demands action.”^4^ The culture leading to burnout begins in medical school and worsens throughout training.^1,5,6,7^ Female resident physicians are disproportionately affected by burnout, likely contributing to the “leaky pipeline” in academic medicine, where women begin as 46% of the workforce yet represent only 23% of full professors and 18% of chairs.^8,9,10,11,12^

Although burnout is well defined, its solution is less clear. Many system-level solutions have been offered; however, neither increased salary, improved electronic medical records, nor reduced hours consistently decrease burnout.^1,7,13^ Individual-level solutions, such as mindfulness, time off, yoga, and structured mentorship, have been offered, and these solutions have mitigated burnout in other fields but have not been similarly effective among physicians.^1,7,14^

Physician burnout likely stems from multiple factors affecting perceptions and habits.^1,15,16^ A narrative review of resident physician burnout cites a perception of stressful work relationships, demanding attending physicians, and a culture in which residents’ needs are inconsequential, correlating with greater burnout.^16^ Protective factors include maintaining optimism and avoiding a mentality of delayed gratification,^16^ suggesting that resident physician perception is a key contributor to burnout.

Professional coaching uses inquiry around perceptions, beliefs, and habits to define, reframe, and align work with personal values.^17,18^ Coaching differs from mentoring, advising, and teaching in that it uses inquisition and metacognition, rather than advice, to help the individual receiving coaching to manage thoughts, feelings, and actions, to move toward fulfillment. Unlike therapy, coaching does not diagnose or clinically treat the individual receiving coaching.^18^ When supported institutionally, coaching is highly accessible and does not require insurance approval or a copay.^17,18,19^

Although coaching is widely used in corporate environments, it is relatively new in academic medicine. Available literature shows that coaching may reduce burnout and improve well-being among physicians and trainees.^17,20,21,22,23^ However, most studies rely on resource-intensive interventions and use variably trained coaches and in-person sessions that are challenging to incorporate and scale within graduate medical education.^20,21,22,23,24^ We posited that a 6-month, web-based group-coaching program led by certified physician coaches would decrease burnout among resident physicians. Here, we describe the results from a pilot randomized clinical trial of our coaching program, Better Together Physician Coaching (hereineafter referred to as Better Together).

## Methods

### Study Design, Setting, and Participants

We piloted a randomized clinical trial of a group life-coaching program, Better Together, with 101 self-reported female resident physicians in graduate medical education at the University of Colorado, a tertiary care center with academic, Veterans Health Administration, safety-net, and community-based settings. All female-identifying University of Colorado residents were eligible to participate and were recruited through email. We initially planned to limit enrollment to 20 participants but received 100 participation requests, so we adjusted our study design to accommodate and analyze efficacy in a pilot randomized clinical trial with a waiting list control group. Information on race and ethnicity was reported by the participants. Participation was voluntary, and all participants provided written informed consent. The intervention occurred between January 1 and June 30, 2021, followed the Consolidated Standards of Reporting Trials (CONSORT) reporting guideline for trial studies,^25^ and was approved by the Colorado Multi-Institutional Review Board. The study protocol is available in Supplement 1.

### Randomization, Allocation Concealment, and Follow-up

**Figure 1.**
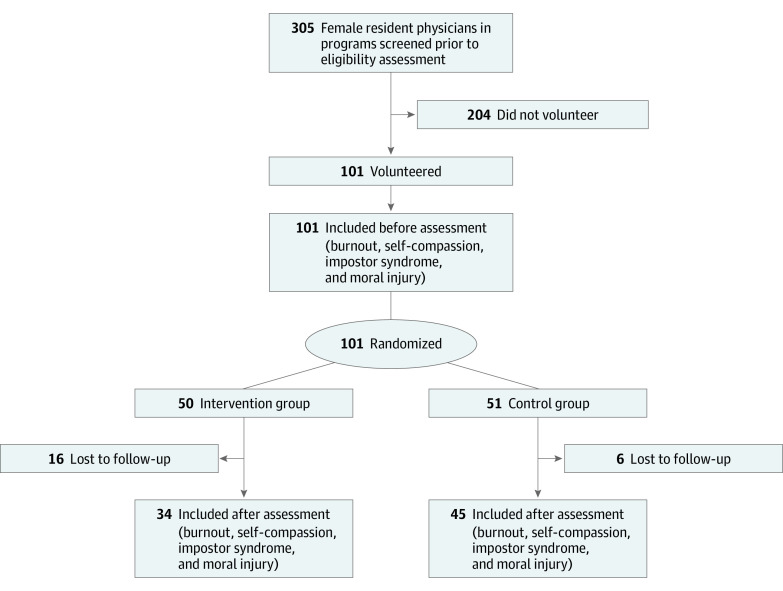
Study Flowchart

Participants were randomly assigned using a computer-generated 1:1 algorithm. Randomization was stratified based on postgraduate year (1, 2, or ≥3) and by specialty: surgical (eg, general surgery and obstetrics and gynecology) vs nonsurgical specialty (eg, internal medicine and pediatrics). Participants were offered a baseline (prior to randomization) and 6-month (end of intervention) survey (Figure 1).^25^

### Description of Intervention

Better Together, a 6-month, web-based group-coaching program, was developed by 2 internal medicine physicians and professional life coaches (T.F. and A.M.). The coaches were certified by The Life Coach School, a thought-based coaching institution with training in both group and individual coaching.^18,19,20^ The Better Together curriculum was housed on a members-only, password-protected website. Participants could participate in any or all of the following: (1) 2 group-coaching calls per week scheduled on weekdays at 7 pm on a video-conferencing platform where up to 5 participants could be coached live on any topic (these calls were recorded to allow for later asynchronous viewing), (2) unlimited anonymous written coaching in an “Ask for Coaching” forum where participants could submit a narrative reflection and receive a written coaching response published on the website, and (3) weekly self-study modules (videos and worksheets) on topics including goal setting, growth mindset, receiving critical feedback, impostor syndrome, and perfectionism. Program facets are outlined in Table 1 and described in detail in the eAppendix in Supplement 2. In instances where coaches supervised participants in a clinical setting, the coaches recused themselves from participant assessment (including serving on the clinical competency or promotions committee) to avoid conflict of interest.

**Table 1.** Components of Better Together Physician Coaching

Live coaching calls	“Ask for coaching” written forum	Self-study
**Method**		
Video-conference platform (Zoom), webinar style; participants request to be brought up for live coaching in front of the group by the “raise hand” function within the platform.	Participants may submit a written request for coaching around any topic. Coaches respond in writing on the website forum for all participants to view within 2 business days.	25 Weekly video modules with 25 accompanying worksheets available on secure, members-only website
**Frequency**		
Two 1-h calls/wk (except on orientation or conclusion or holiday weeks), Tuesday or Thursday 7-8 pm local time	Unlimited submissions 24/7	Unlimited access to video modules and self-study worksheets
**Use of coaching calls**		
45 Coaching calls 121 Unique participant sessions Range, 1-5 participant sessions/call Mean (SD), 2.3 (1.0) participant sessions/call 27 Participants requested coaching sessions, 23 did not Range, 1-13 sessions/participant among those who did request Mean (SD), 4.3 (3.7) sessions/participant among those who did request Median, 3 sessions/participant among those who did request	34 Submissions	Not tracked
**Anonymity**		
To coach		
Optional (if requesting coaching, could turn off video and change name to “anon”); option to come to live calls just to watch others and not raise hand to be coached, which is completely anonymous given webinar style of Zoom	No	Yes
To participants		
Same as above	Yes	Yes

### Study Groups

Participants randomly assigned to the intervention group were offered the coaching program. They were not given protected time to participate and carried the same clinical workload and schedules as participants randomly assigned to the control group. Control group participants received no intervention and were offered the coaching program after the study conclusion (from July to December 2021).

### Study Outcomes

Baseline and end-of-study surveys were administered electronically through the Research Electronic Data Capture system. The survey contained questions on demographic characteristics and validated instruments measuring dimensions of well-being.

#### Primary Outcome: Burnout

Burnout was measured using the Maslach Burnout Inventory (MBI).^26^ The MBI is defined by 3 subscales: (1) emotional exhaustion (EE; feeling emotionally exhausted because of work [9 items]), (2) depersonalization (DP; detached and impersonal treatment of patients [5 items]), and (3) professional accomplishment (PA; beliefs around competence and success at work [8 items]). Each item is a 7-point question on a Likert-type scale. Higher scores on the EE and DP subscales and lower scores on the PA subscale indicate higher burnout. We used the most commonly applied thresholds for the presence of EE (≥27), DP (≥10), and low PA (≤33),^26,27^ and we considered physicians with EE to have at least 1 manifestation of burnout.^26,27,28^ Emotional exhaustion is a key construct in health care–related burnout; in multiple samples of physicians, a 1-point increase in the EE subscale score has been associated with a 7% increase in suicidal ideation and a 5% to 6% increase in major medical errors.^29,30^

#### Secondary Outcomes: Impostor Phenomenon, Self-compassion, and Moral Injury

Secondary outcomes included the Young Impostor Syndrome Scale score,^31^ which is an 8-item measurement of impostor syndrome with yes or no scoring, where a score of 5 or more indicates the presence of impostor syndrome and a score of less than 5 indicates no impostor syndrome. Neff’s Self-Compassion Scale–Short Form^32^ is a 12-item measurement of self-compassion, where higher scores indicate greater self-compassion (scores of 1.0-2.49 are considered to be low, scores of 2.5-3.5 are moderate, and scores of 3.51-5.0 are high). The Moral Injury Symptom Scale–Healthcare Professionals^33^ is a 10-item Likert scale measurement of moral injury, where the higher scores indicate greater moral injury.

### Power Calculation

A sample size of 100 was chosen based on the capacity to provide the intervention to 50 resident physicians. Assuming a conservative assumption of zero correlation between 2 measurements for the same individual, with 80% power (α = .05, 2-sided), a mean (SD) standardized effect size of 0.8 (1.0) was detectable. With the use of SDs for the MBI components from other literature,^34^ this corresponds to detecting a difference in mean (SD) values between the groups of 7.9 (9.8) in EE, 4.8 (6.0) in DP, and 5.1 (6.4) in PA.

### Statistical Analysis

Statistical analysis was conducted using an intention-to-treat analysis. Descriptive statistics were computed for the respondent characteristics overall and by group, with baseline comparisons made using the Wilcoxon rank sum test for continuous covariates and the Fisher exact test or the χ^2^ test for categorical covariates. We similarly compared the characteristics of postsurvey responders with those of nonresponders. To evaluate the intervention effect, we used a linear mixed model to use all available data without excluding the female resident physicians who did not complete follow-up surveys. In this model, we included the main effects of period (baseline vs after intervention), treatment (intervention vs control), and the interaction between period and treatment. The interaction effect represents the difference in the change from baseline to after intervention between the groups.

Owing to the differential follow-up response rates in the treatment groups (88.2% control [45 of 51]; 68.0% intervention [34 of 50]), we performed sensitivity analyses to assess the potential effect of missing follow-up survey data on outcomes. We used multiple imputation to impute the missing scores and a 2-sample *t* test to analyze the difference in the changes in scores between the treatment groups. Multiple imputation by chained equations was performed using 10 imputed data sets, and the imputation model for each score included baseline characteristics, treatment assignment, and baseline score.^35,36^ We also performed a sensitivity analysis in which the baseline score was carried forward for those with missing follow-up scores. All *P* values were from 2-sided hypothesis tests, and statistical significance was assessed at *P* ≤ .05. All analyses were performed using R, version 4.0.4 (R Group for Statistical Computing).

## Results

### Participants

Within 2 weeks of recruitment, 101 female resident physicians from 12 graduate medical education programs at the University of Colorado enrolled in Better Together. All participants completed the baseline survey, and 50 were randomly assigned to the intervention group. By self-report, the mean (SD) age of the participants was 29.4 (2.3) years, all participants identified as cisgender female, most (96 [95.0%]) were heterosexual, and 81 (80.2%) were White. There were 19 resident physicians (18.8%) from surgical specialties, and multiple training levels were represented (Table 2).^31,32,33^ There were no significant differences in baseline characteristics or scale scores between the intervention and control groups. Of the 101 initial participants, 79 responded to the follow-up survey (78.2% response rate). Of those who did not complete the follow-up survey, a higher proportion were participants in the intervention group (72.7% [16 of 22]; *P* = .01). Otherwise, no significant differences were noted in the baseline characteristics or scale scores between those who did and those who did not complete the follow-up survey (eTables 1 and 2 in Supplement 2).

**Table 2.** Participant Characteristics and Scores at Baseline

Characteristic	Overall (N = 101)	Control group (n = 51)	Intervention group (n = 50)	*P* value^a^
Age, y				
Mean (SD)	29.4 (2.3)	29.6 (2.2)	29.1 (2.3)	.20
Median (range)	29.0 (25.0-35.0)	29.0 (26.0-35.0)	29.0 (25.0-35.0)	
Postgraduate year, No. (%)				
1	33 (32.7)	16 (31.4)	17 (34.0)	.96
2	43 (42.6)	22 (43.1)	21 (42.0)	
≥3	25 (24.8)	13 (25.5)	12 (24.0)	
Gender identity				
Cisgender woman	101 (100)	51 (100)	50 (100)	>.99
Transgender woman, cisgender man, transgender man, nonbinary, or other	0	0	0	
Racial and ethnic identity, No. (%)				
Asian	11 (10.9)	5 (9.8)	6 (12.0)	.36
Black	2 (2.0)	2 (3.9)	0	
White	81 (80.2)	39 (76.5)	42 (84.0)	
Other^b^	7 (6.9)	5 (9.8)	2 (4.0)	
Sexual orientation, No. (%)				
Bisexual	3 (3.0)	2 (3.9)	1 (2.0)	.52
Gay or lesbian	2 (2.0)	0	2 (4.0)	
Heterosexual	96 (95.0)	49 (96.1)	47 (94.0)	
Homosexual	2 (2.0)	0	2 (4.0)	
Other queer	0	0	0	
Prefer not to say	0	0	0	
Residency specialty, No. (%)				
Nonsurgical	82 (81.2)	41 (80.4)	41 (82.0)	.84
Surgical	19 (18.8)	10 (19.6)	9 (18.0)	
Primary outcome: burnout				
EE subscale score, mean (SD) (range, 0-54)	27.1 (8.55)	28.2 (8.93)	26.0 (8.10)	.16
DP subscale score, mean (SD) (range, 0-30)	11.0 (5.52)	11.1 (5.61)	10.9 (5.48)	.98
PA subscale score, mean (SD) (range, 0-48)	34.7 (6.41)	33.7 (6.92)	35.8 (5.73)	.25
Secondary outcomes: self-compassion, impostor syndrome, moral injury				
Self-compassion score, mean (SD) (range, 12-60)^c^	33.6 (7.17)	33.0 (8.01)	34.3 (6.21)	.23
Young Impostor Syndrome Scale score, mean (SD) (range, 0-8)^d^	5.40 (2.13)	5.39 (2.17)	5.40 (2.11)	.98
Moral Injury Symptom Scale score, mean (SD) (range, 10-100)^e^	42.2 (11.1)	43.7 (11.7)	40.7 (10.2)	.26

Abbreviations: DP, depersonalization; EE, emotional exhaustion; PA, personal accomplishment.

^a^ Wilcoxon rank sum test, Fisher exact test, and Pearson χ^2^ test.

^b^ American Indian and Alaska Native, Native Hawaiian and Other Pacific Islander, other, including 2 or more races and ethnicities, and prefer not to say.

^c^ Neff’s Self-Compassion Scale–Short Form^32^ measured self-compassion. In this scale, higher scores indicate greater self-compassion.

^d^ The Young Impostor Syndrome Scale^31^ was used to assess the presence of impostor syndrome, where higher values are a greater indication of impostor syndrome. Respondents mark yes or no to 8 questions about how they feel at work. The scale is considered as a dichotomous outcome where responding yes to at least 5 of 8 questions indicates the presence of impostor syndrome.

^e^ The Moral Injury Symptom Scale^33^ is a 10-point scale ranging from strongly disagree to strongly agree. After recoding the positively worded items, a total score is computed, with higher values indicating greater moral injury.

### Engagement

Forty-five 1-hour group-coaching calls occurred during the study. Of 52 potential calls, 5 were not completed owing to holidays, scheduling needs, or recording errors, and 2 were designated “orientation” and “farewell,” respectively, and did not contain coaching content. Over the 45 calls, 27 participants requested and received coaching in 121 unique sessions, each lasting between 10 and 30 minutes. Among the 27 participants who received live coaching, the number of coaching sessions per participant ranged from 1 to 13 (mean [SD], 4.3 [3.7]; median, 3). The mean (SD) number of individuals coached per call was 2.3 (1.0). We received 21 submissions for anonymous written coaching on the Ask for Coaching forum.

### Primary Outcome: Burnout

**Figure 2.**
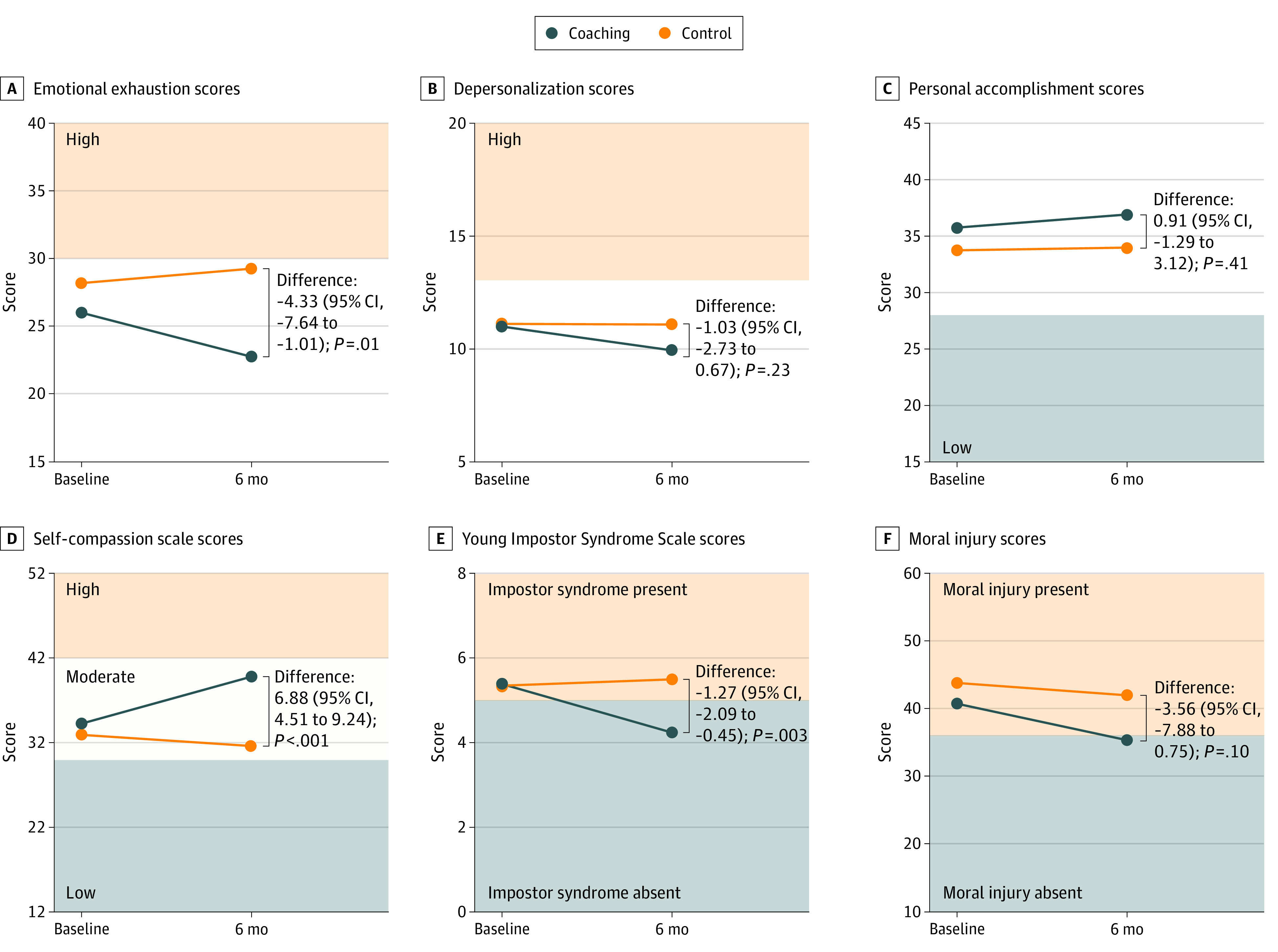
Outcome Results and Estimated Changes in Scores From Baseline to After Intervention A, Emotional exhaustion scores (range, 0-54; lower scores indicate less emotional exhaustion). B, Depersonalization scores (range, 0-30; lower scores indicate less depersonalization). C, Personal accomplishment scores (range, 0-48; higher scores indicate more personal accomplishment). D, Self-compassion scale scores (range, 12-60; higher scores indicate more self-compassion). E, Young Impostor Syndrome Scale scores (range, 0-8; lower scores indicate less impostor syndrome). F, Moral injury scores (range, 10-100; lower scores indicate less moral injury).

There was a significant difference in the change in mean score of the EE subscale of the MBI from baseline to after the intervention between the intervention group and the control group. Participants in the intervention group experienced a reduced mean (SE) EE score, while the control group experienced an increased mean (SE) EE score (−3.26 [1.25] vs 1.07 [1.12]; *P* = .01) (Figure 2; Table 3). Both groups experienced an improvement in mean (SE) DP and PA scores, which was slightly greater in the intervention group; however, this improvement was not statistically significant (mean [SE] scores in intervention group vs control group: DP, −1.06 [0.64] vs −0.03 [0.58]; *P* = .23; and PA, 1.16 [0.83] vs 0.25 [0.75]; *P* = .41).

**Table 3.** Estimated Changes in Scale Scores From Baseline to After Intervention From Mixed-Effects Model

Outcome	Intervention group		Control group		Absolute difference in change, intervention vs control, points (95% CI)	*P* value
	Participants, No.	Estimated change, points (SE)	Participants, No.	Estimated change, points (SE)		
**Primary outcome: burnout**						
EE score						
Baseline	50	–3.26 (1.25)	50	1.07 (1.12)	–4.33 (–7.64 to –1.01)	.01
After intervention	34		44			
DP score						
Baseline	50	–1.06 (0.64)	51	–0.03 (0.58)	–1.03 (–2.73 to 0.67)	.23
After intervention	34		43			
PA score						
Baseline	50	1.16 (0.83)	51	0.25 (0.75)	0.91 (–1.29 to 3.12)	.41
After intervention	34		43			
**Secondary outcomes: self-compassion, impostor syndrome, moral injury**						
Self-compassion score						
Baseline	49	5.55 (0.89)	50	–1.32 (0.80)	6.88 (4.51 to 9.24)	<.001
After intervention	34		43			
Young Impostor Syndrome Scale score						
Baseline	50	–1.16 (0.31)	51	0.11 (0.27)	–1.27 (–2.09 to –0.45)	.003
After intervention	32		44			
Moral Injury Symptom Scale score						
Baseline	50	–5.39 (1.62)	50	–1.83 (1.47)	–3.56 (–7.88 to 0.75)	.10
After intervention	34		43			

Abbreviations: DP, depersonalization; EE, emotional exhaustion; PA, professional accomplishment.

### Secondary Outcomes: Impostor Syndrome, Self-compassion, and Moral Injury

Participants in the intervention group had significantly reduced impostor syndrome symptom scores from baseline, while participants in the control group had increased scores (mean [SE] scores, −1.16 [0.31] vs 0.11 [0.27]; *P* = .003) (Table 3). The intervention group also had improved self-compassion scores compared with the control group (mean [SE] scores, 5.55 [0.89] vs −1.32 [0.80]; *P* < .001). Participants in the intervention group had a greater reduction in moral injury scores compared with participants in the control group, although this difference was not statistically significant (mean [SE] scores, −5.39 [1.62] vs −1.83 [1.47]; *P* = .10). Similar results were obtained using multiple imputation and when baseline scores were carried forward for missing follow-up scores (eTable 3A and 3B in Supplement 2).

## Discussion

In this pilot randomized clinical trial, participants who received coaching had a statistically significant reduction in EE and impostor syndrome scores and showed improvement in self-compassion scores. The magnitude of the reduction in EE scores was substantial and was higher than in previously described wellness interventions.^21,22,24,37,38^ We did not find significant differences in the PA or DP MBI scale scores between the 2 treatment groups, and the differences in moral injury scores did not reach statistical significance. We encountered unanticipated demand for this intervention and demonstrated that coaching in a group setting can address resident physician burnout.

Our findings are consistent with prior coaching studies that showed a positive effect in some but not all aspects of physician well-being^17,21,22,23,24,37,38^ and that support the theory that more than 1 intervention may be necessary to target multiple facets of well-being. A randomized clinical trial of 6 telephone coaching sessions for primary care physicians decreased burnout and increased job satisfaction but did not reduce stress or turnover intention or increase job efficacy.^38^ In a study of medical residents in the Netherlands, 6 face-to-face coaching sessions over 10 months resulted in improved personal resources and reduced burnout symptoms, yet no changes were observed in work engagement or psychological flexibility.^24^ Although previous studies show that group coaching supports physicians’ professional identity formation and healthy work-life integration,^39,40^ these studies did not find an association between group coaching and personal well-being factors, including burnout. The group coaching in these studies had prescriptive content with preselected topics (eg, conflict management)^40^ and a different delivery format (ie, 3 full-day sessions and 5 two-hour sessions over 4 months).^39,40^ In contrast, Better Together’s longitudinal, multimodal coaching format allowed participants to have a self-paced, flexible, and customizable experience.

The Better Together coaching program represents both an institutionally sponsored and individually harnessed tool that encourages systemic commitment and individual responsibility for well-being. Better Together was designed for diverse needs and full schedules; participants could choose which modalities to use based on individual learning styles (written, verbal, or visual), goals, and competing demands. We used group coaching to cultivate a psychologically safe atmosphere in which vulnerability was normalized and traditional hierarchy was discouraged.^40,41^ Group coaching also supported delivery feasibility by maximizing the number of residents who received coaching per session. Although this coaching program was designed before the emergence of the COVID-19 pandemic, no changes to the format or content were required to accommodate the need for virtual participation. This unanticipated strength provided opportunities for connection and support during the pandemic. The Better Together coaching program is unique in the following domains that likely contribute to its success.

### Use of Certified Physician Coaches

The coaches were physicians who understand the challenges of medical training. Many life coaching interventions for physicians do not use certified physician coaches but instead hire external, nonphysician consultants or rely on noncertified volunteer faculty with varying degrees of training in coaching techniques.^17,21,22^

### Asynchronous, Multimodal Content Delivery

Our asynchronous, online model allowed content to be accessed on demand. The repository of recorded calls meant that participants could still benefit even if they were unable to attend live. Residents knew that participation was voluntary and committed to maintaining confidentiality of their peers. Anonymous written coaching allowed participants to be coached in a time and place that worked for them.

### Group Coaching Model

The group format allowed each coach to host one 1-hour call per week and to reach many participants. This wide reach would not have been feasible with a 1:1 model, and participants would not have the benefit of observing peers receiving coaching or the normalization of a culture of authenticity and vulnerability.

### Future Work

Given the need to address burnout and the promising findings of this pilot trial, our goal is to evaluate Better Together at multiple graduate medical education sites nationally. We aim to understand which components of the intervention were most useful; examine the reproducibility of these results with a more diverse group of coaches among a larger and more diverse population of resident physicians; determine the optimal frequency, themes, and duration of coaching; and analyze the durability of the intervention.

### Limitations

This study has some limitations. Given that this pilot randomized clinical trial was at a single institution, our sample size was based on estimated feasibility and thus underpowered to detect a meaningful effect for all outcomes. The voluntary nature of participation may reflect a selection bias toward participants having more distress than nonparticipants; however, it is also possible that others experiencing more burnout were too overwhelmed to volunteer. It is possible that observed outcome improvements accrued, in part, from expectations rather than the intervention itself. We were not funded to deliver an alternative noncoaching intervention to participants in the control group, but such an intervention is a potential way to mitigate this bias in future studies.

Although our sample of participants was demographically representative of female physician residents at the University of Colorado, participation was limited to women, and only 14 participants self-identified as underrepresented in medicine (URM; including race and ethnicity and sexual orientation). Inclusion of women was intentional because they are more affected by burnout. The number of URM participants is small and may limit the applicability of these results to other populations, where even higher rates of burnout are observed. A potential area for bias in recruitment could be in the deficiency of diversity and URM representation among the program leaders, who are both White. Additional trials are needed to explore the efficacy of this model across demographic identities, including racial, ethnic, sexual, and gender minority groups, and across a spectrum of career stages.

It was not feasible to blind the coaches, so they knew the identities of the participants and the participants knew which group they were in. The coaches were University of Colorado faculty members with teaching roles, which provided relatability and credibility but also introduced potential social desirability and selection biases because some participants may have opted to enroll owing to prior experience with the coaches. Both coaches are internal medicine physicians, and their identity was included in the recruitment emails. It is possible that this disclosure affected recruitment of participants from specialties outside of internal medicine and participation in the program. We did not want participants to perceive their participation as “graded” or measured, and therefore we did not measure data on website or material use or coaching call attendance, which may have prevented us from identifying desirability or selection bias.

A significantly higher proportion of residents in the intervention group did not complete the postintervention survey compared with the residents in the control group. This finding could have been due to email fatigue (participants in the intervention group received 2 emails weekly regarding the program), or perhaps the participants in the control group were more motivated to fill out the postintervention survey in anticipation of receiving the intervention. We attempted to assess the effect of missing follow-up survey data on study outcomes with the sensitivity analyses (eTable 3A and 3B in Supplement 2).

Finally, both coaches obtained certification with personal time and funds ($18 000 for a 6-month certification course through the Life Coach School) and were supported with 10% full-time equivalents as conditions of their grant funding for the development of this program. Together, they spent a total of 45 hours in live coaching and 20 hours responding to written coaching over the course of the 6-month intervention. This program could be challenging to scale broadly for those wishing to create a similar program.

## Conclusions

Life coaching for female resident physicians significantly improved EE, self-compassion, and impostor syndrome scores. The Better Together coaching program demonstrated the feasibility of using certified physician coaches to deliver group-based coaching through a multimodal delivery format. This model holds great promise for physician well-being; however, widespread adoption and long-term sustainability will depend on the institutional investment in coaching.

## Notes

Supplement 1.
Trial Protocol

Supplement 2. **eAppendix.** Facets of Better Together Physician Coaching Program **eTable 1.** Participant Characteristics by Follow-up Response **eTable 2.** Scale Scores and Missingness at Baseline and Post-intervention by Intervention Group **eTable 3A.** Missing Data Analysis: Multiple Imputation Results for Changes in Scale Scores Pooled Over 10 Imputed Data Sets **eTable 3B.** Missing Data Analysis: Carry-Forward of Baseline Scores for Those With Missing Follow-up Scores

Supplement 3.
Data Sharing Statement
