# Incomplete Conflict of Interest Disclosures

**DOI:** 10.1001/jamanetworkopen.2022.20348

**Published:** 2022-06-06

In the Original Investigation titled “Effect of a Novel Online Group-Coaching Program to Reduce Burnout in Female Resident Physicians: A Randomized Clinical Trial,”^1^ published on May 6, 2022, the authors’ disclosures of potential conflicts of interest were incomplete. In addition to the disclosures for Drs Fainstad and Mann, there were additional relationships for these authors and 3 coauthors with Better Together Physician Coaching, which was the intervention included in this trial. The complete conflict of interest disclosures statement should have included the following:

“Drs Fainstad and Mann are professional life coaches and coach clients (including physicians) outside of their academic roles in independently owned and operated LLCs; in that capacity, they do not recruit or coach medical trainees. In addition, Dr Fainstad is an unpaid co-founder of Better Together and receives support from the University of Colorado Division of General Internal Medicine; Dr Mann is an unpaid co-founder of Better Together and receives support from a University of Colorado Anschutz Medical Campus (CU) Department of Medicine Program for Academic Clinician Educators (PACE) grant; Ms Shah is a paid research assistant with Better Together funded through a CU Department of Medicine PACE grant and previously was an owner and therapist at PS Counselling LLC (which closed in August 2021); Ms Dieujuste is a paid research assistant with Better Together funded through a CU Department of Medicine PACE grant; and Dr Thurmon is an unpaid professional coach with Better Together and provided paid coaching and consultation to the University of Kansas Department of Surgery in January and February of 2021. No other disclosures were reported.”

The disclosure section of the article has been corrected.^1^
