# Supplementary material for: Effect of a Novel Online Group-Coaching Program to Reduce Burnout in Female Resident Physicians: A Randomized Clinical Trial
Source: JAMA Netw Open. 2022 May 6;5(5):e2210752. doi: 10.1001/jamanetworkopen.2022.10752 (PMC9077483; doi:10.1001/jamanetworkopen.2022.10752)
Supplement: Supplement 1. — Trial Protocol [file jamanetwopen-e2210752-s001.pdf]

Supplement 2. Study Protocol

COMIRB Protocol

COLORADO MULTIPLE INSTITUTIONAL REVIEW BOARD
CAMPUS BOX F-490 TELEPHONE: 303-724-1055 Fax: 303-724-0990

Protocol #: 20-2035

Protocol: Effect of a Novel Online Physician Group-Coaching Program to Reduce Burnout in Trainees: A Randomized Controlled Trial

Hypotheses and Specific Aims: Our hypothesis is that a web-based positive psychology coaching program intervention with female identifying residents will result in: (1) decreased burnout, (2) increased self-compassion, (3) decreased imposter syndrome, (4) decreased moral injury

Aim 1: Develop a pilot web-based, group coaching program for female trainees in the University of Colorado

Aim 2: Evaluate the impact of the intervention

Aim 3: Refine the content and platform to improve the intervention and expand ability to recruit learners.

Research Methods

A. Outcome Measure(s):

We will include measures of primary and secondary outcomes as measured on a pre- and post- program survey including the Maslach Burnout Inventory (MBI), Young Imposter Syndrome Scale (YISS), Moral Injury Symptom Scale (MISS) and Neff Self Compassion Score (SCS).

B. Description of Population to be Enrolled:

This pilot study will recruit individuals from the University of Colorado Graduate Medical Education residency programs. We are limiting the pilot program to female identifying trainees who have greater than 1 year of training left in order to give trainees time to complete the program prior to graduation. Enrollment in the program is entirely voluntary and trainees can cease enrollment at any time. Due to greater than anticipated interest in the program, 101 trainees from 12 training programs on Anschutz Medical Campus were recruited.

C. Study Design and Research Methods

This is a randomized controlled trial. We will recruit, enroll and randomize beginning in November 2020, and start the program in January 2021. Trainees will participate in coaching for 6 months via a web-based system. The website will be secure and only available for program participants. There will be monthly modules (Appendix 1) with asynchronous, self-directed delivery, designed to mitigate the time constraints and faculty development required of traditional in-person coaching. Video recordings of group-coaching calls will be made available to the program participants via the secure website

with the intent to allow participants to benefit from group coaching even if they aren't able to attend the calls live. These recordings will not contain PHI of participants.

The control group will be offered the coaching program after completion of this study.

All participants in both intervention and wait-list/control groups will be invited to complete the quantitative post-program survey in June 2021. At the time of the post-program survey in 6/2021, the wait-list control group will not have received the coaching intervention. Incentive for completion of the post survey is a $25 amazon gift card. Participants in the intervention group will be invited to participate in qualitative interviews. Members of the wait-list control group will not be invited to complete qualitative interviews in June 2021. Incentive for completion of a qualitative interview will be a $75 amazon gift card. Following the quantitative and qualitative data collection, the control group will be offered the 6-month coaching program from July-December 2021.

D. **Description, Risks and Justification of Procedures and Data Collection Tools:** This is a low-risk intervention. Positive psychology coaching is not meant to replace or function as evaluation or medical care. We are using an internally developed survey in addition to multiple validated surveys that reflect the literature in similar programs. Any participant who demonstrates medically concerning issues will be immediately referred to appropriate evaluation. Trainees will be coached by faculty outside their own department when possible. The content of the program is not intended for evaluation, so will not be shared with training programs. The participants will be instructed to maintain confidentiality of their peers' information, although given the group nature of this intervention, confidentiality cannot be assured. The faculty interests in their roles as clinical supervisors and coach are aligned in the promotion of trainee success. Both relationship (clinical preceptor and coach) are in the primary interest of professional and personal development of the trainee. All faculty coaches will recuse themselves of voting in the Clinical Competency Committees (CCC, required by all programs by the ACGME) for trainees in this program. This has precedent specifically in the Internal Medicine program where mentors on the CCC do not vote on their personal mentees, which is the model we will use in our coaching program.

E. **Potential Scientific Problems:**

Due to the nature of the intervention, it is not necessarily possible to randomize or compare groups. If interested enrollment exceeds the capacity of this program, we will be able to randomize the groups for prospective comparison. There is no ability to blind the intervention due to the nature of the program. The evaluation will rely on self-reported outcome measures, although we have made effort to identify and utilize evidence-based and validated evaluation tools. We cannot control for selection bias in how volunteers choose to participate in the program.

F. **Data Analysis Plan:**

Statistical analysis will be performed in an intent-to-treat basis. We will utilize univariate statistics for characterization of the sampled group. Comparisons between the group MBI and SCS over time will be used with paired t-test. Independent group t-test will be used to compare groups if enrollment exceeds capacity, and we are able to randomly generate a control group and intervention group. A PRA will independently analyze the interview transcripts using a mixed inductive/deductive approach to theme analysis supported by ATLAS.ti software.

G. Summarize Knowledge to be Gained:

The purpose of this study is to develop a web-based positive psychology coaching intervention for female physician trainees. The impact of positive psychology coaching in medicine is still in the early stages of research and development, and the specific role and success of this style of coaching in female trainees is unstudied. This specific web-based coaching structure has not been described in medical education literature and is an opportunity for meaning contribution to the body of coaching scholarship in medicine.

H. References:

1. Dyrbye LN, West CP, Satele D, et al. Burnout among U.S. medical students, residents, and early career physicians relative to the general U.S. population. Acad Med. 2014;89(3):443-451.
2. Houkes I, Winants Y, Twellaar M, et.al. Development of burnout over time and the causal order of the three dimensions of burnout among male and female GPs. A three-wave panel study. BMC Public Health. 2011,11:240
3. The State of Women in Academic Medicine 2018-2019 Exploring Pathways to Equity, Association of American Medical Colleges
4. Theeboom T, Beersma B, van Vianen A. Does coaching work? a meta-analysis on the effects of coaching on individual level outcomes in an organizational context. *J Posit Psychol*. 2014;9(1):1-18.
5. Siegler J. Raising physician performance with coaching: respect, collaboration and unified expectations can catalyze future success. *Healthc Exec*. 2016;31(3):88,90-91.

Kyeong, LW.

6. Self-compassion as a moderator of the relationship between academic burn-out and psychological health in Korean cyber university students. Personality and individual Differences. 2013, 54:899-902
7. Ey S, Moffit M, Kinzie JM, et.al. Feasibility of a comprehensive wellness and suicide prevention program: a decade of caring for physicians in training and practice. J Grad Med Educ. 2016;8(5):747-753.
8. Raj, KS. Well-being in residency: a systematic review. J Grad Med Educ. 2016;8(5):674-684

Palamara K, Kauffman C, Chang Y, et.al. Professional Development Coaching for Residents: Results of a 3-year Positive Psychology Coaching Intervention. J Gen Intern Med. 2018, 33(11):1842-4

9. Dyrbye LN, Shanafelt TD, Gill PR, Satele DV, West CP. Effect of a Professional Coaching Intervention on the Well-being and Distress of Physicians: A Pilot Randomized Clinical Trial. JAMA Intern Med. 2019;179(10):1406–1414. doi:10.1001/jamainternmed.2019.2425

10. Hayes, S.C, Strosahl, K.D., & Wilson, K.G. (2012). *Acceptance and commitment therapy: The process and practice of mindful change* (2nd edition). New York, NY: The Guilford Press.
