# Supplementary material for: Effect of a Novel Online Group-Coaching Program to Reduce Burnout in Female Resident Physicians: A Randomized Clinical Trial
Source: JAMA Netw Open. 2022 May 6;5(5):e2210752. doi: 10.1001/jamanetworkopen.2022.10752 (PMC9077483; doi:10.1001/jamanetworkopen.2022.10752)
Supplement: Supplement 2. — eAppendix. Facets of Better Together Physician Coaching Program eTable 1. Participant Characteristics by Follow-up Response eTable 2. Scale Scores and Missingness at Baseline and Post-intervention by Intervention Group eTable 3A. Missing Data Analysis: Multiple Imputation Results for Changes in Scale Scores Pooled Over 10 Imputed Data Sets eTable 3B. Missing Data Analysis: Carry-Forward of Baseline Scores for Those With Missing Follow-up Scores [file jamanetwopen-e2210752-s002.pdf]

Supplementary Online Content

Fainstad T, Mann A, Suresh K, et al. Effect of a novel online group-coaching program to reduce burnout in female resident physicians: a randomized clinical trial. *JAMA Netw Open.* 2022;5(5):e2210752.
doi:10.1001/jamanetworkopen.2022.10752

eAppendix. Facets of Better Together Physician Coaching Program
eTable 1. Participant Characteristics by Follow-up Response
eTable 2. Scale Scores and Missingness at Baseline and Post-intervention by Intervention Group
eTable 3A. Missing Data Analysis: Multiple Imputation Results for Changes in Scale Scores Pooled Over 10 Imputed Data Sets
eTable 3B. Missing Data Analysis: Carry-Forward of Baseline Scores for Those With Missing Follow-up Scores

This supplementary material has been provided by the authors to give readers additional information about their work.

eAppendix. Facets of Better Together Physician Coaching Program

Live Coaching Calls. The live coaching calls were facilitated by one of the two certified physician coaches (TF or AM). Calls were hosted on the Zoom video-conferencing platform and were 60 minutes each. During the calls, getting coached was optional and voluntary: participants could raise their hand to indicate a request to be coached while the other participants on the call observed. Call recordings were saved in a secure folder accessible only to participants so they could view any calls they were not able to attend.

"Ask-for-Coaching" Written Coaching. The secure website housed an online forum where participants could anonymously request coaching via written format. Responses to written coaching requests were posted to the forum within 1-business day by TF or AM. This asynchronous format allowed access to coaching despite the ever-changing and round-the-clock schedules of trainees and provided a kinesthetic learning format for participants to engage with the coaching material. Ask-for-Coaching could be accessed at any point throughout the 6-month intervention, and was completely anonymous; the coachee is not identifiable to other members of BT.

Worksheets. At the start of each month of content, worksheets were released which accompanied each weekly theme (i.e., receiving feedback, setting goals, adopting a growth mindset, or defining work-life balance). These were provided in both a digital and mailed hardcopy format. The worksheets facilitate metacognitive techniques of awareness and processing feelings. The worksheets were voluntary and meant for independent self-study.

Webinars. Short 5–10-minute videos corresponding to weekly content were released at the start of each month. The webinars were chalk-talk style didactics given by AM or TF to introduce our weekly themes. As they may be watched repeatedly, and at any time, the webinars served as an asynchronous component of the program that participants engaged with at their own pace.

© 2022 Fainstad T et al. *JAMA Network Open.*

eTable 1. Participant Characteristics by Follow-up Response

	Overall (N=101)	Complete (N=79)	Baseline only (N=22)	*P* value
**Age (years)**				0.530
Mean (SD)	29.4 (2.26)	29.3 (2.32)	29.5 (2.06)	
**PGY**				0.811
1	33 (32.7%)	25 (31.6%)	8 (36.4%)	
2	43 (42.6%)	35 (44.3%)	8 (36.4%)	
>=3	25 (20.7%)	19 (24.0%)	6 (27.2%)	
**Gender Identity**				1.00
Cis-female	101 (100%)	79 (100%)	22 (100%)	
**Racial/Ethnic background**				0.739
Asian	11 (10.9%)	9 (11.4%)	2 (9.1%)	
Black	2 (2.0%)	1 (1.3%)	1 (4.5%)	
White	81 (80.2%)	63 (79.7%)	18 (81.8%)	
Other* inc. two or more races	7 (6.9%)	6 (7.6%)	1 (4.5%)	
**Sexual orientation**				0.465
Bisexual	3 (3.0%)	3 (3.8%)	0 (0%)	
Heterosexual	96 (95%)	75 (94.9%)	21 (95.5%)	
Homosexual	2 (2.0%)	1 (1.3%)	1 (4.5%)	
Other queer	0 (0%)	0 (0%)	0 (0%)	
Prefer not to say	0 (0%)	0 (0%)	0 (0%)	
**Intervention**				0.014
Control	51 (50.5%)	45 (57.0%)	6 (27.3%)	
Intervention	50 (49.5%)	34 (43.0%)	16 (72.7%)	
**Residency Specialty**				1.00
Non-Surgical	82 (81.2%)	64 (81.0%)	18 (81.8%)	
Surgical	19 (18.8%)	15 (19.0%)	4 (18.2%)	
**PRIMARY OUTCOME: BURNOUT**				
**EE Subscale score (range: 0-54)**				0.724
Mean (SD)	27.1 (8.55)	27.3 (8.89)	26.6 (7.41)	
**DP Subscale score (range: 0-30)**				0.451
Mean (SD)	11.0 (5.52)	11.2 (5.90)	10.4 (4.14)	
**PA Subscale score (range: 0-48)**				0.126
Mean (SD)	34.7 (6.41)	35.3 (6.20)	32.9 (6.82)	
**SECONDARY OUTCOMES: SELF-COMPASSION, IMPOSTER SYNDROME, MORAL INJURY**				
**Total Self-Compassion Score (range: 12-60)**				0.657
Mean (SD)	33.6 (7.17)	33.8 (7.40)	33.0 (6.43)	
**Total Young Impostor Score (range: 0-8)**				0.313
Mean (SD)	5.40 (2.13)	5.29 (2.26)	5.72 (1.67)	
**Total Moral Injury Symptom Score (range = 10-100)**				0.891
Mean (SD)	42.2 (11.1)	42.3 (11.8)	42.0 (8.57)	

*American Indian and Alaska Native, Native Hawaiian and Other Pacific Islander, Other, including two or more races, Prefer not to say

© 2022 Fainstad T et al. *JAMA Network Open.*

eTable 2. Scale Scores and Missingness at Baseline and Post-intervention by Intervention Group

	Control		Intervention	
	**Baseline (N=51)**	**Post-intervention (N=45)**	**Baseline (N=50)**	**Post-intervention (N=34)**
**PRIMARY OUTCOME: BURNOUT**				
**EE Subscale score (range: 0-54)**				
**Mean (SD)**	28.2 (8.93)	29.5 (9.18)	26.0 (8.10)	22.4 (8.21)
**Missing**	1 (2.0%)	1 (2.2%)	0 (0%)	0 (0%)
**DP Subscale score (range: 0-30)**				
**Mean (SD)**	11.1 (5.61)	11.3 (4.86)	10.9 (5.48)	9.79 (5.54)
**Missing**	0 (0%)	2 (4.4%)	0 (0%)	0 (0%)
**PA Subscale score (range: 0-48)**				
**Mean (SD)**	33.7 (6.92)	34.6 (6.94)	35.8 (5.73)	37.3 (6.77)
**Missing**	0 (0%)	2 (4.4%)	0 (0%)	0 (0%)
**SECONDARY OUTCOMES: SELF-COMPASSION, IMPOSTER SYNDROME, MORAL INJURY**				
**Total Self-Compassion Score* (range: 12-60)**				
**Mean (SD)**	33.0 (8.01)	32.1 (7.18)	34.3 (6.21)	39.6 (7.27)
**Missing**	1 (2.0%)	2 (4.4%)	1 (2.0%)	0 (0%)
**Total Young Impostor Score**(range: 0-8)**				
**Mean (SD)**	5.39 (2.17)	5.43 (2.05)	5.40 (2.11)	4.19 (2.21)
**Missing**	0 (0%)	1 (2.2%)	0 (0%)	2 (5.9%)
**Total Moral Injury Symptom Score*** (range = 10-100)**				
**Mean (SD)**	43.7 (11.7)	41.7 (11.2)	40.7 (10.2)	35.6 (10.5)
**Missing**	1 (2.0%)	2 (4.4%)	0 (0%)	0 (0%)

*Neff's Self-Compassion Scale – Short Form (SCS-SF)[30] measured self-compassion. In this scale, higher scores indicate greater self-compassion.
**Young Impostor Syndrome Scale (YISS)[29] was used to assess the presence of imposter syndrome, where higher values are a greater indication of imposter syndrome. Respondents mark "yes" or "no" to 8 questions about how they feel at work. The YISS is considered as a dichotomous outcome where responding "Yes" to at least five of the eight questions indicates the presence of imposter syndrome.
***The Moral Injury Symptom Score (MISS)[31]a 10-point scale ranging from strongly disagree to strongly agree. After re-coding the positively worded items, a total score is computed, with higher values indicating greater moral injury.

© 2022 Fainstad T et al. *JAMA Network Open.*

eTable 3A. Missing Data Analysis: Multiple Imputation Results for Changes in Scale Scores Pooled Over 10 Imputed Data Sets

	**Difference in change, Intervention vs. Control (SE)**	***P* value**
**EE Score**	-4.10 (1.69)	0.02
**DP Score**	-1.29 (0.84)	0.13
**PA Score**	0.92 (1.28)	0.47
**Self-Compassion Score**	6.88 (1.26)	<0.001
**Young Impostor Score**	-1.34 (0.40)	0.001
**Moral Injury Symptom Score**	-3.44 (2.21)	0.13

eTable 3B. Missing Data Analysis: Carry-Forward of Baseline Scores for Those With Missing Follow-up Scores

	**Difference in change, Intervention vs. Control (SE)**	***P* value**
**EE Score**	-2.88 (1.35)	0.04
**DP Score**	-0.58 (0.67)	0.39
**PA Score**	0.66 (0.86)	0.44
**Self-Compassion Score**	5.18 (1.02)	<0.001
**Young Impostor Score**	-0.86 (0.33)	0.01
**Moral Injury Symptom Score**	-2.34 (1.76)	0.19

© 2022 Fainstad T et al. *JAMA Network Open.*
