# Supplementary material for: Effect of a Novel Online Group-Coaching Program to Reduce Burnout in Female Resident Physicians: A Randomized Clinical Trial
Source: JAMA Netw Open. 2022 May 6;5(5):e2210752. doi: 10.1001/jamanetworkopen.2022.10752 (PMC9077483; doi:10.1001/jamanetworkopen.2022.10752)
Supplement: Supplement 3. — Data Sharing Statement [file jamanetwopen-e2210752-s003.pdf]

# Data Sharing Statement

Fainstad. Effect of a Novel Online Group-Coaching Program to Reduce Burnout in Female Resident Physicians. *JAMA Netw Open*. Published May 06, 2022.
doi:10.1001/jamanetworkopen.2022.10752

**Data**
**Data available:** No
